# Integrative analyses of proteomics and RNA transcriptomics implicate mitochondrial processes, protein folding pathways and GWAS loci in Parkinson disease

**DOI:** 10.1186/s12920-016-0164-y

**Published:** 2016-01-21

**Authors:** Alexandra Dumitriu, Javad Golji, Adam T. Labadorf, Benbo Gao, Thomas G. Beach, Richard H. Myers, Kenneth A. Longo, Jeanne C. Latourelle

**Affiliations:** 1 Department of Neurology, Boston University School of Medicine, Boston, MA 02118 USA; 2 Proteostasis Therapeutics Inc., Cambridge, MA 02139 USA; 3 Banner Sun Health Research Institute, Sun City, AZ 85351 USA; 4 Genome Science Institute, Boston University School of Medicine, Boston, MA 02118 USA

**Keywords:** Parkinson disease, RNA-Seq, MS3 proteomics, GWAS loci, Data integration, Metallothioneins, Mitochondria, Protein folding pathways

## Abstract

**Background:**

Parkinson disease (PD) is a neurodegenerative disease characterized by the accumulation of alpha-synuclein (SNCA) and other proteins in aggregates termed “Lewy Bodies” within neurons. PD has both genetic and environmental risk factors, and while processes leading to aberrant protein aggregation are unknown, past work points to abnormal levels of SNCA and other proteins. Although several genome-wide studies have been performed for PD, these have focused on DNA sequence variants by genome-wide association studies (GWAS) and on RNA levels (microarray transcriptomics), while genome-wide proteomics analysis has been lacking.

**Methods:**

This study employed two state-of-the-art technologies, three-stage Mass Spectrometry Tandem Mass Tag Proteomics (12 PD, 12 controls) and RNA-sequencing transcriptomics (29 PD, 44 controls), evaluated in the context of PD GWAS implicated loci and microarray transcriptomics (19 PD, 24 controls). The technologies applied for this study were performed in a set of overlapping prefrontal cortex (Brodmann area 9) samples obtained from PD patients and sex and age similar neurologically healthy controls.

**Results:**

After appropriate filters, proteomics robustly identified 3558 unique proteins, with 283 of these (7.9 %) significantly different between PD and controls (q-value < 0.05). RNA-sequencing identified 17,580 protein-coding genes, with 1095 of these (6.2 %) significantly different (FDR *p*-value < 0.05); only 166 of the FDR significant protein-coding genes (0.94 %) were present among the 3558 proteins characterized. Of these 166, eight genes (4.8 %) were significant in both studies, with the same direction of effect. Functional enrichment analysis of the proteomics results strongly supports mitochondrial-related pathways, while comparable analysis of the RNA-sequencing results implicates protein folding pathways and metallothioneins. Ten of the implicated genes or proteins co-localized to GWAS loci. Evidence implicating SNCA was stronger in proteomics than in RNA-sequencing analyses.

**Conclusions:**

We report the largest analysis of proteomics in PD to date, and the first to combine this technology with RNA-sequencing to investigate GWAS implicated loci. Notably, differentially expressed protein-coding genes were more likely to not be characterized in the proteomics analysis, which lessens the ability to compare across platforms. Combining multiple genome-wide platforms offers novel insights into the pathological processes responsible for this disease by identifying pathways implicated across methodologies.

**Electronic supplementary material:**

The online version of this article (doi:10.1186/s12920-016-0164-y) contains supplementary material, which is available to authorized users.

## Background

Parkinson disease (PD) is the second most common neurodegenerative disease after Alzheimer’s disease, with approximately six million cases diagnosed worldwide [1]. PD is characterized clinically by impairment of motor function and cognitive abilities. The main neuropathological hallmarks of PD are protein aggregates termed “Lewy Bodies”, which are found within neurons and neuronal processes, and contain many proteins, but consist primarily of the alpha-synuclein protein (SNCA). Multiple neurotransmitters are implicated in PD, with degeneration of dopaminergic neurons in the *substantia nigra* most prominently seen. The disease is considered a “complex disorder” resulting from both environmental and genetic factors. Familial monogenic forms of PD have been identified, with the most common of these attributed to mutations in the leucine-rich repeat kinase 2 (*LRRK2*) [2], Parkin (*PARK2*) [3] and Glucosidase, Beta, Acid (*GBA*) [4] genes, and with several rare monogenic forms recognized, including rare mutations in the alpha-synuclein gene (*SNCA*) [1, 5]. Recently, a large meta-genome-wide association study (GWAS) for PD identified twenty-four loci implicated in disease risk [6, 7], although most of the genes responsible for risk at these loci remain unknown. While few environmental factors have been unequivocally identified for PD [8, 9], the heterogeneity of genetic and environmental factors that contribute to disease etiology makes the understanding of the underlying pathogenic processes difficult to define.

Although protein aggregation is fundamental to PD pathogenesis, and the roles of abnormal protein folding, trafficking and clearance in PD are widely discussed, few studies have sought to investigate disrupted protein levels using genome-wide methods. To our knowledge, the study by Riley et al. [10] is the only past study to perform both RNA-sequencing (RNA-Seq) and proteomics (Liquid chromatography tandem-mass spectrometry) in brain samples for three PD cases and three controls in the striatum and cortex. That study implicated oligodendrocyte function and synaptic vesicle release as disease-related processes, and reinforced the power of genome-wide technologies even for small sample sizes. Thus, while a few high-throughput genome-scale experiments have been performed in the past for PD, including cDNA microarrays [10, 11] and genome-wide association studies (GWAS) [6, 7], the biology of the disease remains poorly understood.

**Fig. 1:**
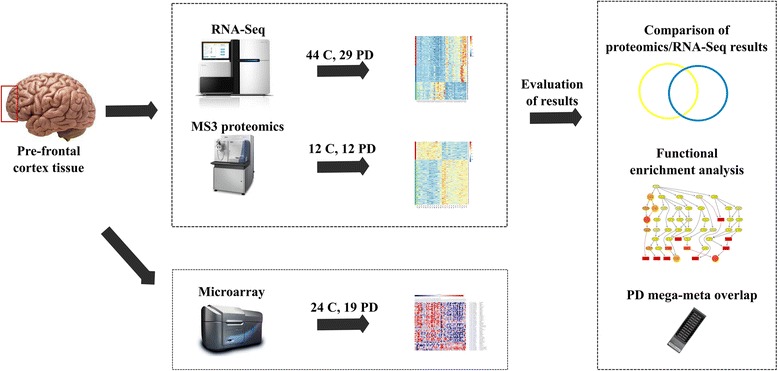
Study overview. Human prefrontal cortex Brodmann Area 9 tissue was used to assess mRNA abundance levels for protein-coding genes using Illumina RNA-Seq, and protein abundance levels using MS3 proteomics in control and PD samples. Differential mRNA and protein abundance analyses were performed to identify lists of differentially abundant genes and proteins, and these top results were evaluated for functional enrichment, as well as for overlap with genes in the vicinity of significant SNPs from a recent mega-meta analysis of PD genome-wide association studies [7]. A subset of the brain samples included in the RNA-Seq study had been previously assessed for mRNA abundance using Agilent microarray technology [11], and the results obtained with the two techniques were compared

While the concordance of proteomics and RNA transcriptomics is known to be weak [12, 13], PD is recognized as a protein aggregation disorder; therefore, the analysis of protein levels in PD brain may provide novel insights in the disease pathology. Our primary interest in this study was to assess the role of disrupted protein homeostasis and to compare this characterization to that measured by RNA quantification technologies. To this end, we applied two state-of-the-art genome-wide technologies, three-stage Mass Spectrometry Tandem Mass Tag (MS3) Proteomics and RNA-sequencing (RNA-Seq) transcriptomics, which were analyzed together with PD GWAS implicated loci and microarray cDNA transcriptomics. The technologies applied for these studies are outlined in Fig. 1 and were performed in a set of overlapping prefrontal cortex samples obtained from male PD patients and sex and age similar neurologically healthy controls.

We first compared the levels of proteins and protein-coding mRNAs between control and PD samples, and then examined both data sources for enriched or common biological signals. The goals of this work were to: 1) assess differences between PD and control brain tissue across multiple biological processes using current genome-wide methodologies, 2) compare disease-related changes in protein and mRNA levels observed in overlapping sets of brain samples, and 3) use the obtained results to aid in interpretation and assessment of prior PD GWAS studies to elucidate the genes that may be responsible for PD risk at the loci implicated by these studies. To our knowledge, no past study has combined these technologies in a series of PD and control brain samples.

## Methods

### Brain samples

Frozen brain tissue from the prefrontal cortex Brodmann Area 9 was available from three brain banks: the National Brain and Tissue Resource for Parkinson’s Disease and Related Disorders at Banner Sun Health Research Institute (BSHRI), Sun City, Arizona, the Harvard Brain Tissue Resource Center McLean Hospital (HBTRC), Belmont, Massachusetts, and the Human Brain and Spinal Fluid Resource Center VA (HBSFRC), West Los Angeles Healthcare Center, California. A total of 29 PD and 44 control samples were included in the RNA-Seq study. Twelve of the 29 PD and 12 of the 44 control samples (selected from those with lowest post-mortem intervals) were included in the proteomics study. Additionally, 19 PD and 24 control samples assayed via RNA-Seq were studied previously in an expression microarray analysis [11]. All control and PD brain samples were derived from males of European ancestry (determined by ancestry informative genotyping [6]) and were assessed for Alzheimer’s disease (AD) pathology. For this assessment, information about plaques and tangles from neuropathology reports was used to exclude cases with any AD-type pathology beyond normal signs of aging, as previously described [14] (see Table 1 and Additional file 1: Table S1). Age at death, post-mortem interval (PMI) and RNA integrity number (RIN) were evaluated for differences between groups and assessed as potential confounders in the analyses.

**Table 1:** Summary statistics for brain samples

Analysis	Description	Control	PD	t-test p
RNA-Seq	Number of samples	44	29	-
	Age at death, years (range)	70.00 (46–97)	77.55 (64–95)	4.6E-3
	PMI, hours (range)	14.36 (2–32)	11.14 (1–31)	0.170
	RIN (range)	7.85 (6.0-9.1)	7.07 (5.8-8.5)	5.9E-5
Proteomics^a^	Number of samples	12	12	-
	Age at death, years (range)	79.50 (61–97)	76.83 (64–88)	0.539
	PMI, hours (range)	5.66 (2–18)	4.16 (2–11)	0.455
Microarray	Number of samples	24	19	-
	Age at death, years (range)	74.75 (58–97)	79.57 (64–94)	0.123
	PMI, hours (range)	13.08 (2–26)	7.57 (2–31)	3.9E-2

^a^All samples studied by proteomics have both RNA-sequencing and microarray transcriptomics

### Processing of samples for proteomics

Prefrontal cortex tissue (50–80 mg) from 12 control and 12 PD samples was homogenized with an ice-cold lysis buffer composed of 8 M urea, 75 mM NaCl, 50 mM Tris, pH 8.2, 1 mM NaF, 1 mM β-glycerophosphate, 1 mM sodium orthovanadate, 10 mM sodium pyrophosphate, 1 mM PMSF and one tablet of protease inhibitors cocktail per 10 mL. The lysate was centrifuged at 20,000 × *g* for 10 min to pellet cellular debris. The protein content of the supernatant was measured using the BCA assay. Protein disulfide bonds were reduced with DTT, and cysteine residues were alkylated with iodoacetamide as previously described [15]. The protein lysates were subjected to a methanol-chloroform precipitation, and then digested overnight with Lys-C (Wako) at a 1/100 enzyme/protein ratio in a buffer comprised of 4 M urea and 50 mM Tris–HCl, pH 8.8. The digest was acidified with formic acid to a final pH of ~2–3, and subjected to C_18_ solid-phase extraction (Sep-Pak, Waters). Isobaric labeling of the peptides was accomplished by dissolving 0.8 mg of 6-plex TMT reagents (Thermo Scientific) in 40 μL acetonitrile, and adding 10 μL of this solution to 100 μg of peptides dissolved in 100 μL of 50 mM HEPES, pH 8.5. These samples were fractionated using strong cation exchange chromatography [15], and the fractions were analyzed by liquid chromatography-MS3 on an LTQ Orbitrap Velos mass spectrometer, as previously described [16]. Each of the four 6-plex proteomics experiments included 3 PD and 3 control samples. The sample processing and data generation for the proteomics experiment were performed by the Gygi Lab (https://gygi.med.harvard.edu/), at Harvard Medical School.

### Database correlation and statistical analysis of proteomic data

All MS/MS and MS/MS/MS spectra were searched against the human IPI database (Version 3.87) from the European Bioinformatics Institute by using the SEQUEST algorithm [17]. Modifications were permitted to allow for the detection of oxidized Met (+16), carboxyamidomethylated Cys (+57), and phosphorylated Ser, Thr and Tyr (+80). All peptide matches were initially filtered based on Xcorr and dcorr scores [15]. A total of 6569 proteins described by IPI IDs were identified in at least one of the 6-plex experiments, with 3600 proteins common to all four 6-plex experiments; only these common 3600 proteins were considered for downstream analyses. After removing the proteins corresponding to IPI IDs that could not be mapped to a proper official gene symbol (4 instances) and averaging the abundance values for proteins with IPI IDs corresponding to the same gene symbol (35 instances), a number of 3558 unique genes were included in the analysis of the proteomics experiment. The mapping of IPI IDs to official gene symbols was performed in April 2014.

The normalization and statistical analysis of the proteomics data were implemented in R. Two steps were used to normalize the raw data: 1) an intra-experimental (within plex) variation step – for each sample, each protein’s raw signal was normalized to the total ion intensity of the protein within the plex, and 2) an inter-experimental (across plexes) variation step – for each sample, each protein’s normalized value from step 1) was transformed by dividing it to the mean of all normalized values of the protein obtained in step 1) of all 24 samples. The Surrogate Variable Analysis (SVA) method implemented in the sva R package [18] (v3.10.0) was used to eliminate latent noise in the data not explained by the categorical factors, including batch effects, by building a set of covariates constructed directly from the proteomics dataset. Three significant surrogate variables (SVs) were identified for the proteomics data (shown in Additional file 1: Table S1). The limma R package [19] was used for statistical analysis of differential abundance, using a linear model fit including the 3 SVs for the contrast between the PD and the control groups. The p-values were adjusted for multiple comparisons using the q-value method [20]; a gene was considered to be significantly different between PD and control samples if it had an adjusted q-value < 0.05. For the full set of differential protein abundance results, see Additional file 2: Table S2.

### RNA extraction

RNA tissue (10–20 mg) for each brain sample included in the sequencing study was homogenized in TRIzol (Invitrogen, Carlsbad, CA). Total RNA was isolated using the Qiagen RNeasy Mini Kit (Qiagen Sciences Inc., Germantown, MD) and further purified using Agencourt RNA Clean magnetic beads (Beckman Coulter, Inc.). The quantity and purity of the RNA was determined by absorbance at 260 nm and by 260/280 absorbance ratio, respectively. Each of the total RNA preparations was individually assessed for RNA quality based on the 28S/18S ratio and the RNA integrity number (RIN) was measured on an Agilent 2100 Bioanalyzer system using the RNA 6000 Nano LabChip Kit (Agilent, Foster City, CA).

### RNA-Seq library preparation and sequencing

For each brain sample, 1 μg RNA was used to construct sequencing libraries using Illumina’s TruSeq RNA Sample Prep Kit. The unmodified manufacturer protocol was followed: mRNA molecules were polyA selected, chemically fragmented, randomly primed with hexamers, synthesized into cDNA, 3′ end-repaired and adenylated, sequencing adapter ligated and PCR amplified. Each adapter-ligated library contained one of twelve TruSeq molecular barcodes. Multiplexed samples were equimolarly pooled into sets of 3 samples per flowcell lane and sequenced using 2x101 ntd paired-end runs on Illumina’s HiSeq 2000 system at the Tufts University sequencing core facility (http://tucf-genomics.tufts.edu/). Demultiplexing and FASTQ file generation (raw sequence read plus quality information in Phred format) were accomplished using Illumina’s Consensus Assessment of Sequence and Variation (CASAVA) pipeline.

### RNA-Seq data processing

Sequenced data were aligned to the UCSC human reference genome (build hg19) using TopHat version 2.0.1 [21] with the following explicit parameters: −−mate-inner-dist = 50, −−mate-std-dev = 50, −−splice-mismatches = 1, −−max-multihits = 20, −−read-mismatches = 3, −−read-edit-dist = 3, −−microexon-search, −−coverage-search. The alignment BAM files were evaluated with the RSeQC quality control python package [22], including assessment of mapping statistics (bam_stat.py script), gene body coverage (geneBody_coverage.py script), GC content of reads (read_GC.py script), and sequence quality based on Phred score (read_quality.py script). There were no outliers detected, and all samples were carried forward in the analysis.

### mRNA gene abundance estimation

Gene expression quantification was performed using htseq-count version 0.5.3p9 (http://www-huber.embl.de/users/anders/HTSeq/doc/count.html), the GENCODE version 17 annotation gtf file (http://www.gencodegenes.org/releases), and the alignment BAM files. The intersection_nonempty mode and the unstranded library type (−−stranded = no option) were specified as parameters for the htseq-count run, with all other options left as default.

### Gene differential expression analysis using DESeq2

For the PD/control differential expression evaluation, we considered the 20,331 protein-coding genes from the GENCODE annotation file. The R (https://www.r-project.org/) package DESeq2 version 1.4.0 [23, 24] was used to perform the differential expression analysis. First, a low-count filter was applied to the protein-coding genes, removing those with 0 counts in more than 50 % of the PD or more than 50 % of the control samples. 17,580 genes passed this filtering criterion. For the differential expression analysis in DESeq2, the instructions from the package vignette were followed. First, a DESeqDataSet object was constructed including as covariates age at death, post-mortem interval (PMI) and binned RNA Integrity Number (two categories: RIN < =7 or >7). The DESeq wrapper was run with default options, and the results function was run specifying independentFiltering = F as an option. The significant genes were considered to be those with a False Discovery Rate (FDR)-adjusted p-value below 0.05, using the procedure of Benjamini and Hochberg [24]. The official symbols of the protein-coding genes were last updated in April 2014, using a custom Matlab script.

For the full set of differential mRNA abundance results, see Additional file 3: Table S3. For a set of genes/proteins with common signal in the RNA-Seq and proteomics experiments (multiple comparison corrected significance in one of the two experiments and nominal significance in the other), see Additional file 4: Table S4.

### Microarray analysis

Nineteen PD and 24 control samples from the ones included in the RNA-Seq study were previously studied as part of an Agilent microarray analysis (44 K One-Color Agilent 60-mer Whole Human Genome Microarray) performed by our investigators [11]. We ran a PD/control differential expression analysis for the microarray data of these 43 samples, following the same protocol described previously [11], with the exception that RIN was binned into two categories, RIN < =7 or >7 (see Additional file 5: Table S5 for results). This analysis change was made to match the analysis from the RNA-Seq study.

### GO and MSigDB enrichment calculation

Enrichment analyses were implemented using the Gene Ontology (GO) annotation database [25] and the Molecular Signatures Database (MSigDB v4.0) [26]. Only the “C2 Canonical Pathways” gene sets were used from the MSigDB database (http://www.broadinstitute.org/gsea/msigdb/index.jsp). To insure that gene sets derived from the strongest implicated genes were not diluted by more weakly implicated genes, we performed enrichment analysis on the top 25 genes, then on the top 50, 100, 350, 600, 850, and 1095 genes obtained from the RNA-Seq analysis and on the top 25, 50, 100, and 283 proteins obtained from the proteomics analysis. GO term enrichment was performed using topGO [27], with the “weight01” algorithm and the “fisher” statistic, after having removed GO terms with less than 10 annotated genes (“nodeSize = 10”). All analyzed RNA-Seq genes and all analyzed proteomics genes were utilized as the background for their respective analyses. Custom scripts in the R statistical environment (http://www.r-project.org/) were created to run the analysis. Enrichment of MSigDB Canonical Pathways gene lists was performed with custom R scripts using the “fisher.test” and “p.adjust” routines. Further processing of enrichment results was performed using custom scripts to generate plots in python with matplotlib [28], ipython notebook [29], and pandas [30].

An identical approach was implemented for the functional enrichment analysis of the 77 genes/proteins with multiple comparison corrected significance in one of the two experiments and nominal significance in the other experiment. For the full set of enrichment analysis results, see Additional file 6: Table S6.

### PD Mega-Meta GWAS Genes

551 SNPs were found as genome-wide significant (*p* < 5E-8) in a recent mega-meta analysis of PD genome-wide association studies [7]. We defined SNPs in a range of 100 kb of a gene to be within the primary regulatory region of the gene, in which 98 % of *cis*-acting eQTLs are likely to occur [31]. Using this criterion, we used Annovar [32] for the annotation of 166 genes identified as potentially under the regulatory control of these 551 genome-wide significant SNPs. These 166 genes were evaluated in terms of differential RNA or protein abundance in our study (see Table 2 and Additional file 7: Table S7).

**Table 2:** Significant genes with evidence from GWAS analysis

Mega-meta GWAS loci^a^	Implicated gene	Description	Additional evidence	Potential PD-relevant biological functions^b^
No rs# available (chr4:816756) intronic	*CPLX1*	complexin 1	Proteomics	Synaptic vesicle exocytosis
rs2263418 (chr12:40582993) upstream	*SLC2A13*	solute carrier family 2 (facilitated glucose transporter), member 13	Proteomics	N/A
rs356182 (chr4:90626111) downstream	*SNCA*	synuclein, alpha (non A4 component of amyloid precursor)	Proteomics	Presynaptic signaling and membrane trafficking
rs8118008 (chr20:3168166) downstream	*SLC4A11*	solute carrier family 4, sodium borate transporter, member 11	RNA-Seq	N/A
rs4889620 (chr16:31131174) intronic	*KAT8*	K(lysine) acetyltransferase 8	RNA-Seq	Histone acetyltransferase activity, transcription factor binding
rs6812193 (chr4:77198986) intronic	*FAM47E*	family with sequence similarity 47, member E	RNA-Seq	Transcription cofactor activity
rs823118 (chr1:205723572) upstream	*NUCKS1*	nuclear casein kinase and cyclin-dependent kinase substrate 1	RNA-Seq	N/A
rs1375131 (chr2:135954797) UTR3	*ZRANB3*	zinc finger, RAN-binding domain containing 3	RNA-Seq	DNA annealing helicase and endonuclease activities
rs11724635 (chr4:15737101) upstream	*CD38*	CD38 molecule	RNA-Seq	Signal transduction, calcium signaling
rs34195153 (chr1:154913723) downstream	*PYGO2*	pygopus family PHD finger 2	RNA-Seq	Signal transduction

^a^Since some genes have multiple SNPs within 100 kb of their start and end sites showing association to PD status at p-values < 5E-8 [7], only the SNP with the lowest p-value is shown for each implicated gene. SNP coordinates are based on the hg19 (GRCh37.p13) human reference. SNP annotations: downstream/outstream = outside the gene boundaries, within 100 kb from the start or end site of the gene, intronic = located in the gene intron, UTR3 = located in the 3′-UTR of the gene

^b^Based on gene information from GeneCards (http://www.genecards.org/), accessed in January 2015

## Results

### Proteomics study

**Fig. 2:**
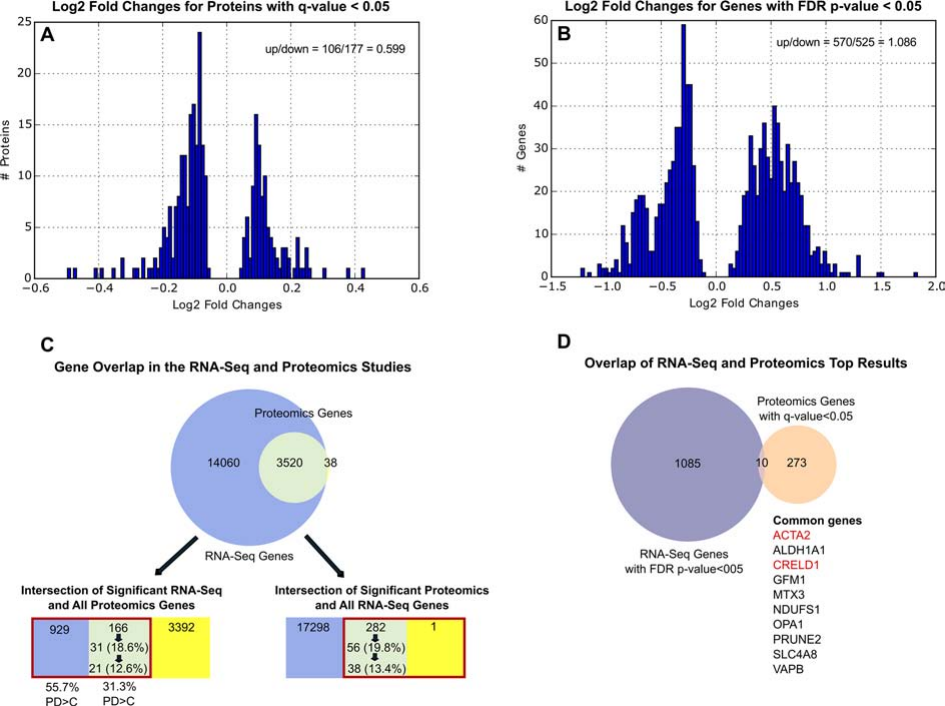
Description of top results for the RNA-Seq and MS3 proteomics experiments. Panels **a** and **b** display the distribution of log2 fold change values for the results with multiple comparison corrected significance obtained in proteomics (*N* = 283) and RNA-Seq (*N* = 1095) differential abundance analyses. The number of protein-coding genes assayed in the RNA-Seq experiment was almost five times larger than the number of proteins assayed in the MS3 proteomics experiment. Panel **c** summarizes the overlap between the genes analyzed by RNA-Seq and proteomics, either for all genes from the two experiments (upper part of panel **c**) or for the genes with multiple comparison corrected significance in one experiment and all genes from the other (lower part of panel **c**). The colors of the diagram sections indicate genes present only in the RNA-Seq experiment (blue), genes present in both the RNA-Seq and the proteomics experiments (green), and genes present only in the proteomics experiment (yellow). The brown boxes highlight the genes with multiple comparison corrected significance – either 1095 for RNA-Seq or 283 for proteomics. For the lower part of panel **c**, the green sections display two additional numbers (with percentages). These numbers represent the number of genes with multiple comparison corrected significance in one study and nominal significance in the other (first number), and the number of genes with multiple comparison corrected significance in one study, nominal significance in the other, and same direction of effect between the two studies (second number). Below the diagram corresponding to the “Intersection of Significant RNA-Seq and All Proteomics Genes” header, two additional percentages are displayed; they represent the percentages of genes with increased mRNA abundance in PD compared to control for the 1) 929 genes with multiple comparison corrected significance in the RNA-Seq study and not present in the proteomics study, and 2) 166 genes with multiple comparison corrected significance in the RNA-Seq study and present in the proteomics study (the two sections of the diagram directly above). Only 10 genes were in common between the RNA-Seq and proteomics results with multiple comparison corrected significance, with eight of them showing the same direction of effect and two of them (highlighted in red) showing opposite direction of effect, as displayed in panel **d**

A total of 6569 unique proteins were identified across the four 6-plex sample sets used for the proteomics study, with 3558 unique proteins detected in all four sets and used in the analysis of differential abundance. 283 proteins were significantly different between PD and control samples after adjustment for multiple comparisons (q-value < 0.05), with 106 of these proteins (37.5 %) showing increased levels and 177 showing decreased levels in PD brain compared to control brain (see Fig. 2 and Additional file 2: Table S2 for details).

**Fig. 3:**
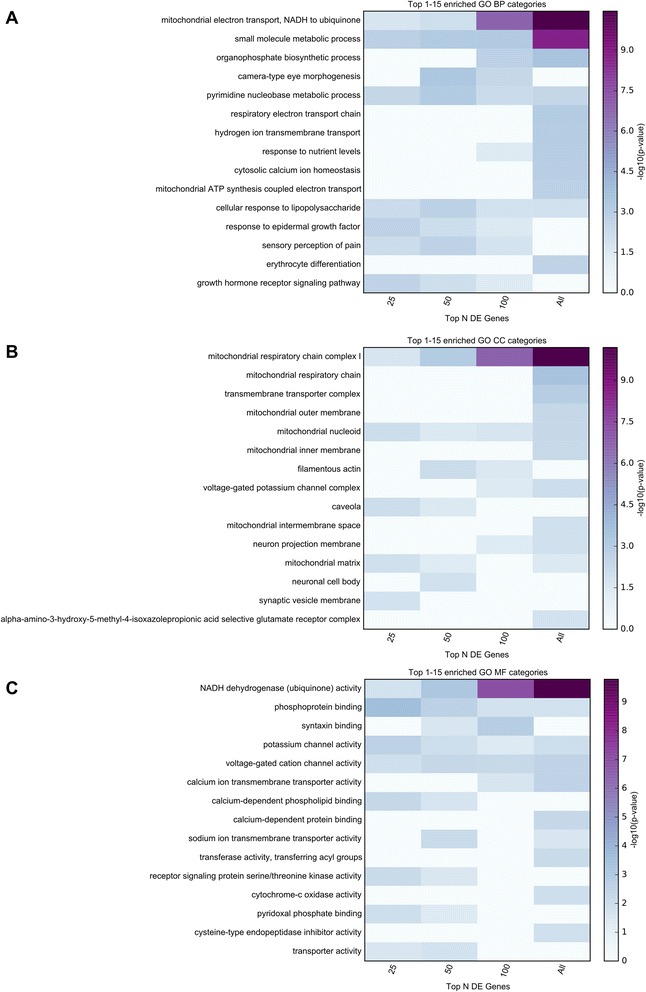
TopGO enrichment analysis results for the proteomics study. Functional enrichment analysis was performed for 4 subsets of the significant results obtained in the MS3 proteomics study (top 25, 50, 100, all 283 genes with multiple comparison corrected significance). The top 15 enriched GO terms belonging to GO Biological Processes (BP), GO Cellular Components (CC), and GO Molecular Functions (MF) are displayed in panels **a**, **b**, and **c**, respectively. The presented top results are those with the smallest p-values in any of the subsets of genes used. The top category for each process implicates mitochondrial function as impaired in PD. DE = differentially expressed

**Fig. 4:**
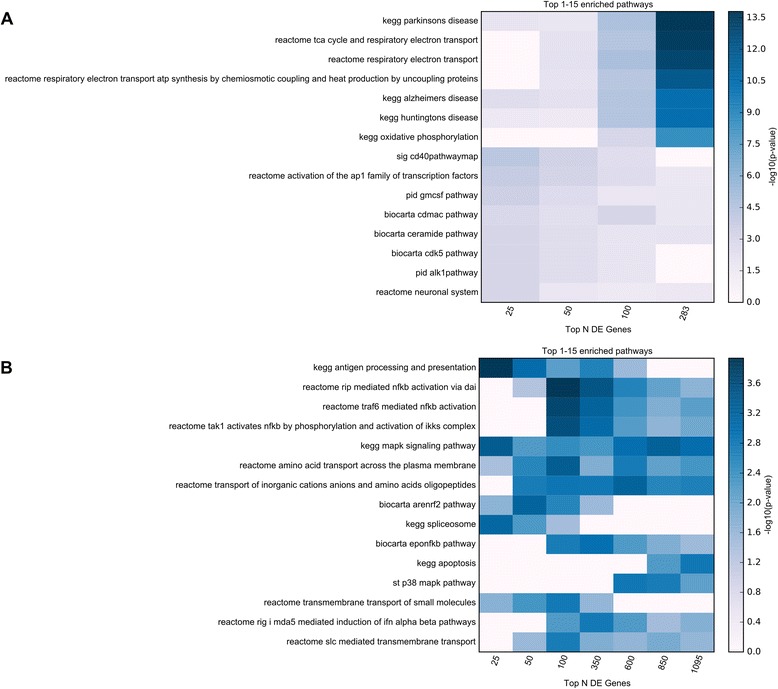
MSigDB canonical pathway enrichment analysis results. Functional enrichment analysis was performed for **a** 4 subsets of the significant genes obtained in the proteomics study (top 25, 50, 100, all 283 genes with multiple comparison corrected significance) and **b** 7 subsets of the significant genes from the RNA-Seq study (top 25, 50, 100, 350, 600, 850, all 1095 genes with multiple comparison corrected significance). The top 15 enriched MSigDB Canonical Pathways for RNA-Seq and MS3 proteomics results are displayed in panels **a** and **b**, respectively. The presented top results are those with the smallest p-values in any of the subsets of genes used. DE = differentially expressed

Functional enrichment analyses were implemented using GO categories and Broad Institute’s Molecular Signature Database (MSigDB) canonical pathways. To insure that functional sets derived from the strongest implicated proteomics genes were not diluted by more weakly implicated genes, the enrichment was performed for all 283 significant proteomics genes, as well as for subsets of these genes (e.g. top 25, 50, 100 genes). Top enrichment results are visualized in Figs. 3 and 4. Detailed enrichment results for all GO terms and MSigDB canonical pathways significant for any of the used subsets of significant genes are available in Additional file 6: Table S6.

The top Biological Processes category was “mitochondrial electron transport, NADH to ubiquinone” (*p* = 3.53E-11), the top Cellular Component category was “mitochondrial respiratory chain complex I” (*p* = 6.39E-11), and the top Molecular Function category was “NADH dehydrogenase (ubiquinone) activity” (*p* = 1.63E-10). Similarly, the top MSigDB Canonical Pathway was “REACTOME RESPIRATORY ELECTRON TRANSPORT”. These findings provide consistent and compelling evidence that an agnostic assessment of genome-wide proteomics analysis strongly supports impaired mitochondrial function as fundamental to PD pathogenesis.

### mRNA sequencing study

#### Differentially abundant protein-coding genes

We compared the mRNA abundance of 17,580 protein-coding genes (passing a low-count filter) of 29 PD to 44 control prefrontal cortex samples (see Table 1 for sample description). A total of 1095 genes (6.2 %) were identified with significantly different mRNA levels at FDR *p*-values < 0.05, with 570 of these genes (52.1 %) showing increased RNA level in PD compared with controls (Fig. 2 and Additional file 3: Table S3).

**Fig. 5:**
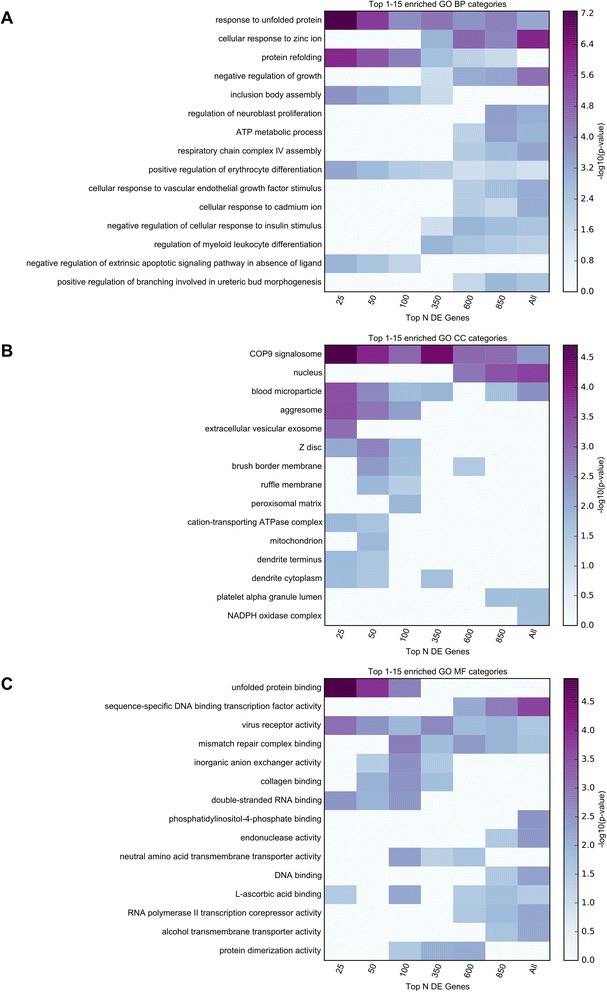
TopGO enrichment analysis results for the RNA-Seq study. Functional enrichment analysis was performed for 7 subsets of the FDR-significant genes obtained in the RNA-Seq study (top 25, 50, 100, 350, 600, 850, all 1095 genes with multiple comparison corrected significance). The top 15 enriched GO terms belonging to GO Biological Processes (BP), GO Cellular Components (CC), and GO Molecular Functions (MF) are displayed in panels **a**, **b**, and **c**, respectively. The presented top results are those with the smallest p-values in any of the subsets of genes used. The Cellular components category “COP9 signlaosome” (CSN) has not been previously implicated in PD. DE = differentially expressed

Functional enrichment analyses were performed in a similar manner to those described for the proteomics results, both for the full set of FDR-significant genes, as well as for subsets of these genes at increasingly stricter gene-sample cutoffs (e.g. top 25, 50, 100, 350, 600, 850 genes). The top 15 MSigDB canonical pathways, and the top 15 enriched terms for each GO set used (Biological Processes, Cellular Components, and Molecular Functions) identified using any of the considered subsets from the FDR-significant genes are displayed in Figs. 4 and 5. Detailed enrichment results are available in Additional file 6: Table S6.

Both the top Biological Processes category “response to unfolded protein” and the top Molecular Function category “unfolded protein binding” implicate responses to abnormal protein structure, which, although not seen in the proteomics analysis, is not surprising given the abnormal protein aggregation seen in PD. The top Cellular Components category “COP9 signalosome” (CSN) has not been previously implicated in PD. The CSN is a conserved multiprotein complex, which mainly functions in the control of proteolysis [33]. The CSN has also been implicated in the control of NF-kappaB in innate immunity, as well as T-cell activation and maturation. Similar to the Cellular Components result above, the MSigDB implicates the “KEGG ANTIGEN PROCESSING AND PRESENTATION” and “REACTOME RIP MEDIATED NFKB ACTIVATION VIA DAI”, providing support for a role of inflammation in PD. In addition, seven metallothionein genes out of 19 present in the gene family showed significantly increased abundance in PD relative to control samples.

### Overlap of RNA-Seq and proteomics analyses

A set of 3520 genes was common between the 17,580 protein-coding genes derived from the RNA-Seq study and the 3558 unique proteins derived from the MS3 proteomics study. However, of the 1095 FDR-significant genes from the RNA-Seq analysis, only 166 were available in the proteomics dataset, while out of the 283 significantly differentially abundant proteins (q-value < 0.05), one was missing from the RNA-Seq dataset (see Fig. 2, panel c).

Given the different rate of detection in the proteomics study for the RNA-Seq FDR significant genes (where only 15.1 % were characterized by MS3 proteomics) compared to the remaining genes (where 20.2 % were characterized by MS3 proteomics), we asked whether or not there was an observed difference in terms of direction of effect between the 166 RNA-Seq FDR significant genes detected via proteomics compared to the remaining 929 undetected via proteomics. Interestingly, the genes with increased expression in PD compared to control at FDR-level of significance were more likely than expected by chance to be undetected in the proteomics study. This result was highly significant (χ^2^ = 33.68, *p* = 1E-8), with 52/166 (31.3 %) genes detected by proteomics showing increased expression in PD, compared with 518/929 (55.7 %) genes not detected by proteomics showing increased expression in PD. This result could indicate a potential post-transcriptional regulatory mechanism that prevents protein translation for specific highly expressed mRNAs involved in disease-related processes. This potential mechanism might also be one reason for the limited overlap between the proteomics and the RNA-Seq measurements, which restricts the analysis of overlapping pathways between the two studies.

When comparing the genes significant after adjustment for multiple comparisons from the two datasets, only ten of them were identified by both analyses, with eight showing the same direction of effect (these ten genes are displayed in Fig. 2, panel d). However, among the 283 genes with q-value < 0.05 in the proteomics analysis, 56 were observed to also have nominal significance in the mRNA analysis, and 38 of these also had the same direction of effect. Among the 166 proteins present in the 1095 FDR-significant RNA-Seq genes, 31 had nominal significance, and 21 of these also had the same direction of effect. In total, 77 unique genes showed evidence for PD involvement from both the gene expression and the protein abundance experiments (see Additional file 4: Table S4 for details). As these genes may be of particular interest in functional studies of PD, we performed functional enrichment for them (Additional file 6: Table S6, tabs “Common Genes GO” and “Common Genes MSigDB CP”). The Cellular Component term “neuron projection membrane” (*p* = 0.0019) is the strongest category identified in this analysis, with the Biological Process category “superoxide metabolic process” (*p* = 0.0036) and the Molecular Function category “protein N-terminus binding” (*p* = 0.0046) among the top findings.

To assess the relationship between RNA expression dysregulation and relative protein abundance, the results for the mRNAs and for the proteins were compared for the 3520 common genes. Weak, but significant correlation for fold changes (Spearman rank correlation: *r* = 0.075, *p* = 7.4E-6) was observed. The correlation for fold changes increased when examining the sets of significant genes after adjustment for multiple comparisons from the RNA-Seq study (*N* = 166, Spearman rank correlation: *r* = 0.185, *p* = 0.016) or from the proteomics study (*N* = 282, Spearman rank correlation: *r* = 0.142, *p* = 0.016).

**Fig. 6:**
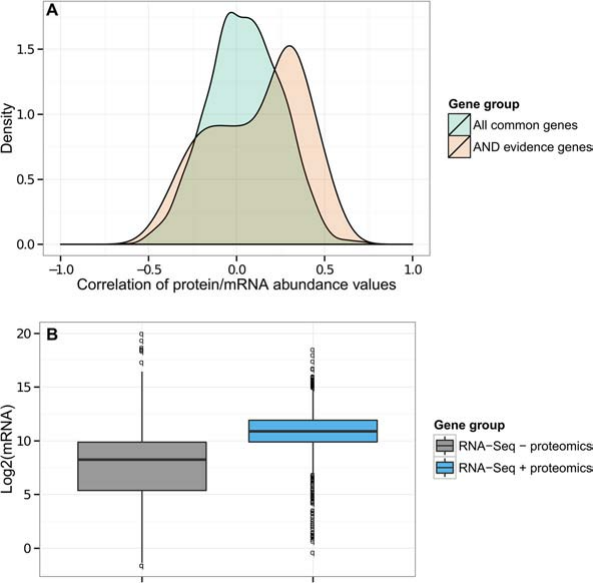
Combined assessment of mRNA and protein abundance results. **a** Comparison of 1) the protein abundance/mRNA abundance correlations for all 3520 genes common between the RNA-Seq and proteomics studies (“All common genes”, displayed in green) and 2) the protein abundance/mRNA abundance correlations for the 77 genes with common signal in both experiments (“AND evidence genes”, displayed in orange). A gene was considered to show common signal if it had multiple comparison corrected significance in one of the two experiments and nominal significance in the other experiment. As it can be observed from the density of the correlations, the “AND evidence genes” showed stronger correlation between the mRNA and the protein levels. **b** Comparison of the mRNA abundance of 1) genes available in the RNA-Seq study, but not in the proteomics study (“RNA-Seq - proteomics”, grey color) and 2) genes available in both the RNA-Seq and the proteomics studies (“RNA-Seq + proteomics”, blue color). The figure displays the log2 values of the genes’ mean mRNA abundance in the 24 samples common between the RNA-Seq and the proteomics analyses. The mRNA abundance of the genes captured by proteomics is significantly increased compared to those missed by MS3 proteomics measurements. This result indicates a potential detection bias for the MS3 proteomics technique for proteins translated from highly expressed mRNAs

To further evaluate the relationship between mRNA and protein abundance in the 24 samples with common data, we looked at the differences in terms of absolute correlation values between protein/mRNA abundance for all common genes as opposed to 1) the 77 common genes with joint evidence from the full RNA-Seq and the proteomics studies (“AND evidence genes”, with multiple comparison corrected significance in one study and nominal significance in the other) and 2) the 438 common genes with evidence from either the full RNA-Seq study or the proteomics study (“OR evidence genes”, with multiple comparison corrected significance in either study). While there was no significant difference between the absolute correlation values of all genes and those belonging to the “OR evidence genes” (t-test *p*-value = 0.18), there was a significant difference between the absolute correlation values of all genes and those belonging to the “AND evidence genes” (t-test *p*-value = 9.3E-6, Fig. 6).

### Application to PD GWAS gene-discovery

Identifying the specific genes responsible for risk at GWAS loci is often difficult, as the regions where SNPs show significant levels of statistical association can span multiple genes and the gene closest to the SNP with strongest p-value is not necessarily the one responsible for association. Thus, an important goal of this project was to provide a resource for the evaluation and prioritization of results from other PD-related high-throughput studies, such as genetic variants from genome-wide association (GWA) studies. The recent mega-meta analysis of PD GWA studies [7] identified over 500 genetic variants in 24 loci (see Methods, PD Mega-Meta GWAS Genes). Using the criterion of genes within 100 kb of a GWAS SNP, within which 98 % of *cis*-acting eQTLs with primary regulatory effects are likely to occur [31], 166 genes were identified (Additional file 7: Table S7). Ten of these genes, displayed in Table 2, were present among our study’s significant RNA-Seq (FDR *p* < 0.05) or proteomics (q-value < 0.05) results.

Notably, SNCA showed significantly increased protein levels in the proteomics analysis, but did not show altered RNA levels in the RNA-sequencing analysis. This finding supports the view that SNCA protein levels are altered in PD, and this observation may contribute to its accumulation in Lewy bodies.

### Comparison of RNA-Seq and microarray abundance analysis results

Data available from a cDNA Agilent microarray study recently performed by our group to compare gene expression levels of 27 PD to 26 control prefrontal cortex samples [11] offered a medium to validate the RNA-Seq results using an alternative technology. 895 of the genes included in the microarray analysis overlapped with the 1095 FDR-significant genes from the RNA-Seq study. For these genes, significant correlation of fold changes was observed (Spearman rank correlation: *r* = 0.490, *p* < 2.2E-16), with 702 genes (78.43 %) showing the same direction of effect between the two studies. 232 of the 702 genes (33.04 %) also had nominal significance.

Given that a subset of 24 controls and 19 PD samples assayed in the RNA-Seq study were also evaluated in the microarray study [11], we could perform a more direct comparison of differential abundance results obtained across the two technologies (see Methods, Microarray Analysis). Although the RNA for the 43 commonly studied brain samples came from different tissue extractions, both studies were performed in the prefrontal cortex (Brodmann Area 9). For these samples, there were 14,115 protein-coding genes commonly analyzed; among these genes, 80 reached FDR-level of significance in the RNA-Seq analysis. The differential abundance results obtained for the two studies showed significant, albeit low correlation with regard to fold change (Spearman rank correlation: *r* = 0.188, *p* < 2.2E-16). However, the correlation for fold changes was stronger when only the 80 protein-coding genes with FDR *p* < 0.05 in the RNA-Seq analysis were considered (Spearman rank correlation: *r* = 0.471, *p* **=** 3.6E-8). While only 9 of these 80 genes (11.25 %) had nominal significance in the microarray, 62 (77.5 %) showed a consistent direction of effect.

## Discussion

While the pathological hallmark of PD is the accumulation of alpha-synuclein (SNCA) and other proteins in Lewy Bodies, past genome-wide studies have focused on GWAS analyses of DNA sequence variants and RNA transcriptomics by microarray. We believe this is the first investigation of PD brain samples which involves genome-wide analyses for (1) MS3 proteomics, (2) RNA-sequencing transcriptomics, (3) PD GWAS implicated loci, and (4) microarray transcriptomics. All samples studied by proteomics were also studied by RNA-Seq and expression microarrays, and all samples were Brodmann 9 prefrontal cortex.

MS3 proteomics identified 3558 unique proteins, while RNA-sequencing identified 17,580 protein-coding genes and 3520 of these were in common between the two platforms. While 283 proteins and 1095 mRNAs were significantly different between PD and controls after adjustment for multiple comparisons, only eight genes were in common and with the same direction of effect between these two sets of top results.

Our analyses revealed evidence implicating a large number of genes, proteins, and biological networks, including many previously reported in PD, as well as some not previously appreciated for this disease. Among the differentially abundant proteins with prior evidence for PD involvement were SNCA (Synuclein, Alpha (Non A4 Component Of Amyloid Precursor); increased in PD) [34, 35], GAD1 (glutamate decarboxylase 1 (brain, 67 kDa); decreased in PD) [36], and NPTX2 (neuronal pentraxin II; decreased in PD) [37]. Functional enrichment analyses of the proteomics results (Additional file 6: Table S6) strongly support the involvement of mitochondrial-related pathways, as has been previously suggested [10, 38, 39], justifying renewed attention to this area of investigation for PD. Other top enriched biological pathways for the proteomics data point towards neurodegenerative diseases (“KEGG PARKINSONS DISEASE”; “KEGG ALZHEIMERS DISEASE”; “KEGG HUNTINGTONS DISEASE”), suggesting that pathological processes seen in PD may overlap with those seen in other neurodegenerative diseases involving aberrant protein aggregation. Evidence for overlap across multiple neurodegenerative diseases is seen much more strongly in the proteomics analysis than in the RNA-Seq analysis described below, suggesting that proteomics analysis may offer important insights for contrasts across neurodegenerative diseases.

The 1095 genes with FDR-level of significance in the RNA-Seq analysis included several previously associated with PD, such as *SMOX* (spermine oxidase; increased in PD) [11], *SPR* (sepiapterin reductase (7,8-dihydrobiopterin:NADP+ oxidoreductase); increased in PD) [40], *DRD3* (dopamine receptor D3; decreased in PD) [41], and *SYT11* (Synaptotagmin XI; decreased in PD) [42]. Concordant with prior studies describing dysregulation of metallothioneins in PD [10, 43], we observed seven metallothionein genes out of the 19 present in this gene family (http://www.genenames.org/cgi-bin/genefamilies/set/638) showing significantly increased abundance in PD samples compared with controls (*MT1A*, *MT1E*, *MT1F*, *MT1G*, *MT1M*, *MT1X*, *MT2A*; See Additional file 3: Table S3). While the number of genes precludes discussion of all of them, it is worth noting that metallothioneins may have neuroprotective properties [44] and protect against oxidative stress [45].

For the functional enrichment analyses of RNA-Seq results, the top GO terms include protein folding (GO:0006986, “response to unfolded protein”; GO:0042026, “protein refolding”; GO:0051082, “unfolded protein binding”), cellular response to metal ions (GO:0071294, “cellular response to zinc ion”; GO:0071276, “cellular response to cadmium ion”), mitochondrial processes (GO:0046034, “ATP metabolic process”; GO:0008535, “respiratory chain complex IV assembly”), and the ubiquitin conjugation pathway (GO:0008180, “COP9 signalosome”). In addition, the enriched MSigDB canonical pathways strongly support processes and pathways related to immune function (“REACTOME RIP MEDIATED NFKB ACTIVATION VIA DAI”; “KEGG ANTIGEN PROCESSING AND PRESENTATION”; “KEGG MAPK SIGNALING PATHWAY”; See Additional file 6: Table S6), which were not prominent in the proteomics analysis. Given that considerable recent work points to the involvement of neuroinflammatory response in PD [46–48], our findings substantiate that MS3 proteomics and RNA-sequencing provide differing insights for PD.

Analyses comparing the 895 genes included in our previously published microarray analysis [11] that overlapped with the 1095 FDR-significant genes from the RNA-Seq study revealed a significant correlation of fold changes (*p* < 2.2E-16). Of these genes, 702 (78.43 %) showed the same direction of effect between the two studies, indicating that the majority of genes implicated by RNA-Seq show fold-change concordance by microarray technology.

Notably, functional enrichment analysis showed pathways common to both the mRNA and the protein datasets. They included terms related to mitochondria processes and oxidative stress (GO:0005739, “mitochondrion”; “BIOCARTA ARENRF2 PATHWAY”), immune response (GO:0043330, “response to exogenous dsRNA”; GO:0034162, “toll-like receptor 9 signaling pathway”; “BIOCARTA FMLP PATHWAY”; “BIOCARTA CDMAC PATHWAY”), regulation of gene expression (“BIOCARTA PPARA PATHWAY”), brain-related processes (“REACTOME NEUROTRANSMITTER RELEASE CYCLE”), and cell-membrane transport (GO:0035725, sodium ion transmembrane transport”). Enrichment of similar pathway categories to those described above were observed when the 77 genes with joint evidence for the RNA-Seq and proteomics analyses were investigated (Additional file 6: Table S6, “Common Genes GO” and “Common Genes MSigDB CP” tabs).

We observed a significant increase in protein/mRNA correlation for genes with signal at both the mRNA and protein levels, albeit the association was modest (mean Pearson correlation values = 0.25) (Fig. 6). Given that prior studies have reported protein and mRNA levels to be weakly correlated, even in large datasets [49], the absence of strong correspondence between RNA-Seq and MS3 proteomics analyses may be a consequence of the complexity of the mRNA-to-protein signal conversion. RNA-Seq evaluates not only the subset of mRNAs meant for translation, but also mRNAs that are still in the nucleus, mRNAs processed for storage, and mRNAs targeted for degradation. Additionally, while protein abundance levels are influenced by their corresponding mRNA levels, they are also a product of translational and post-translational mechanisms [50], which may further limit the relationship between these two molecules.

Since detection biases for specific types of protein classes are known to exist when protein abundance is measured via proteomics methods [51], we used the 24 samples profiled in the proteomics study to investigate if the mRNA abundance levels of the proteins characterized by MS3 proteomics were different than the mRNA abundance levels of all genes quantified by RNA-Seq analysis. Interestingly, the mRNAs corresponding to proteins detected in the proteomics study showed significantly increased levels when compared to mRNA levels for all protein-coding genes in the RNA-Seq study (t-test *p*-value < 2.2E-16, see Fig. 6). This finding suggests a detection bias towards proteins encoded by genes with highly abundant mRNAs, implying that the RNA-Seq method might have a larger dynamic range of detection than the MS3 proteomics method in human brain tissue.

Although twenty-four PD risk loci have been identified by GWAS studies, the genes responsible for risk at these loci are generally unknown. We overlapped our MS3 proteomics and RNA-Seq top results with loci from a recent mega-meta GWAS analysis by Nalls et al. [7]. Utilizing all SNPs with genome-wide significant p-values identified in the Nalls study, we mapped genes positioned within 100 kb of these SNPs. Using this criterion, 166 genes were identified as positioned near a GWAS-implicated SNP (Additional file 7: Table S7). Ten of these genes showed overlap with the RNA-Seq or proteomics genes with multiple comparison corrected significance (see Table 2). Notably, the SNCA protein is identified as increased in PD by the MS3 proteomics analysis, suggesting that proteomic analysis may lend insights into identifying the risk associated genes at GWAS implicated loci.

The correspondence between protein and mRNA levels defined by proteomics and RNA-sequencing has traditionally been found to be weak, as has been reported in previous work [52]. Indeed, our study did not find strong concordance between differentially abundant protein-coding mRNAs (contrast between 29 PD and 44 control samples) and proteins (contrast between 12 PD and 12 control samples) in prefrontal cortex tissue, with only eight genes present in both studies with multiple comparison corrected significance and same direction of effect. This observation can be attributed to a combination of factors, including: 1) the different number of samples analyzed in the two studies, 2) the approximate five-fold size difference between the genes/proteins with robust signals captured via RNA-Seq and MS3 proteomics techniques, and 3) the detection bias observed for the proteomics technique for proteins with higher mRNA abundance. Although the correspondence between MS3 proteomics-derived protein levels and RNA-Seq-derived RNA levels appears low, these initial attempts at resolving the discrepancies among these platforms offer some novel insights into the pathogenesis of PD.

The fold changes observed in the proteomics data were small, and potential reasons that might contribute to this are: 1) the 3600 proteins included in the proteomics analysis were biased towards abundant proteins that could be robustly identified and variations in abundant proteins tend to be smaller, 2) while the isotopic labeling proteomics techniques, such as tandem-mass tag (TMT) and isobaric tag for relative and absolute quantification (iTRAQ), are mass spectrometry techniques with good quantitative accuracy, quantitation at the peptide level does lead to systematic underestimation of protein fold changes [53], 3) prefrontal cortex is not the primary brain region affected in PD, and 4) proteins in the brain have slower turnover compared with proteins from other tissue types [54]. While age at death was not significantly different between the PD and control groups in the proteomics study, the RNA-Seq PD samples showed a significantly later age at death when compared with the control samples (t-test *p*-value = 0.0046). Additionally, while post-mortem interval values were not significantly different between PD and control samples in the proteomics and RNA-Seq studies, they were significantly increased in the control group from the microarray validation study (t-test *p*-value = 3.9E-2). These are consequences of the fact that human brain tissue with high quality RNA is not easily accessible, especially from neurologically healthy control samples. A final limitation is the omission of non-coding RNAs, which have yielded intriguing findings in previous studies [55]. Because the purpose of this study was to integrate a differential analysis of proteomics data with RNA-sequencing data, we focused exclusively on the analysis of protein-coding genes. However, the analysis of other RNA biotypes represents a logical next step.

The RNA-Seq and proteomics results made publicly available through this study (GEO: GSE68719) are a valuable resource for the evaluation of existing and future high-throughput studies in PD.

## Conclusions

We report the largest analysis of proteomics in PD to date, and the first to combine this technology with both RNA-sequencing and GWAS implicated loci. Combining multiple genome-wide platforms offers novel insights into the pathological processes responsible for this disease by identifying pathways implicated across methodologies. Functional enrichment analysis of the proteomics results strongly supports mitochondrial-related pathways, while comparable RNA-sequence analysis implicated protein folding pathways and metallothioneins. Ten of the implicated genes or proteins co-localized to GWAS loci. Evidence implicating SNCA was stronger in proteomics than in RNA-sequencing analyses.

Our studies emphasize the importance for continuing to expand the numbers of proteins that can be studied by MS3 and related developing technologies for genome-wide proteomics analysis. In addition, as the quantification for larger numbers of specific protein isoforms expands, it is important to expand upon methods to assess the relationship of specific RNA transcripts to specific protein isoforms.

## Additional files

Additional file 1: Table S1. Detailed information for the entire set of 29 PD cases and 44 controls studied by MS3 proteomics, RNA-Seq, and expression microarray. (XLSX 59 kb)

Additional file 2: Table S2. Results for the protein abundance comparison between 12 PD and 12 control samples for the 3558 proteins from the MS3 proteomics study. (XLSX 336 kb)

Additional file 3: Table S3. Results for the mRNA abundance comparison between 29 PD and 44 control samples for the 17,580 protein-coding genes from the RNA-Seq study. (XLSX 2337 kb)

Additional file 4: Table S4. Proteomics and RNA-Seq results for the 77 genes/proteins with common evidence (multiple comparison corrected significance in one of the experiments and nominal significance in the other). (XLSX 58 kb)

Additional file 5: Table S5. Results for the mRNA abundance comparison between 19 PD and 24 control samples present in the microarray expression dataset E-MTAB-812 from ArrayExpress and also included in the RNA-Seq study. (XLSX 3194 kb)

Additional file 6: Table S6. This file contains ten tables: (1) Proteomics GO BP: GO Biological Pathways that showed an enrichment p-value smaller than 0.05 in any of the performed analyses for the proteomics results (using the top 25, 50, 100, or 283 genes with multiple comparison corrected significance); (2) Proteomics GO CC: GO Cellular Components that showed an enrichment p-value smaller than 0.05 in any of the performed analyses for the proteomics results; (3) Proteomics GO MF: GO Molecular Functions that showed an enrichment p-value smaller than 0.05 in any of the performed analyses for the proteomics results; (4) Proteomics MSigDB CP: MSigDB Canonical Pathways that showed an enrichment p-value smaller than 0.05 in any of the performed analyses for the proteomics results; (5) RNA-Seq GO BP: GO Biological Pathways that showed an enrichment p-value smaller than 0.05 in any of the performed analyses for the RNA-Seq results (using the top 25, 50, 100, 350, 600, 850, or 1095 genes with multiple comparison corrected significance); (6) RNA-Seq GO CC: GO Cellular Components that showed an enrichment p-value smaller than 0.05 in any of the performed analyses for the RNA-Seq results; (7) RNA-Seq GO MF: GO Molecular Functions that showed an enrichment p-value smaller than 0.05 in any of the performed analyses for the RNA-Seq results; (8) RNA-Seq MSigDB CP: MSigDB Canonical Pathways that showed an enrichment p-value smaller than 0.05 in any of the performed analyses for the RNA-Seq results; (9) Common Genes GO: GO terms that showed an enrichment p-value smaller than 0.05 in the 77 genes with common evidence in the RNA-Seq and proteomics results (multiple comparison corrected significance in one experiment and nominal significance in the other one); (10) Common Genes MSigDB CP: MSigDB Canonical Pathways that showed an enrichment p-value smaller than 0.05 in the 77 genes with common evidence in the RNA-Seq and Proteomics results. (XLSX 110 kb)

Additional file 7: Table S7. MS3 proteomics and RNA-Seq PD/control comparison results for the 166 genes that were present within 100 kb (based on Annovar annotation) of the 551 genome-wide significant SNPs identified in the Nalls et al. study [7]. When the proteomics or mRNA dataset does not contain a particular gene, “NA” values are displayed in the corresponding columns. (XLSX 62 kb)

## Abbreviations

BP: Biological processes
CC: Cellular components
CSN: COP9 signalosome
eQTL: Expression quantitative trait locus
FDR: False discovery rate
GO: Gene ontology
GWAS: Genome-wide association study
IPI: International protein index
KEGG: Kyoto encyclopedia of genes and genomes
mRNA: Messenger RNA
MS3: Three-stage mass spectrometry tandem mass tag
MSigDB: Molecular signatures database
MF: Molecular functions
PD: Parkinson disease
PMI: Post-mortem interval
RIN: RNA integrity number
RNA-Seq: mRNA sequencing
SNCA: Alpha-synuclein
SNP: Single nucleotide polymorphism
SVA: Surrogate variable analysis
